# Links Between Perceptions of Successes, Problems and Health Outcomes Among Adult Chinese Children: The Mediating Role of Perceptions of Parents’ Feelings and Intergenerational Relationships

**DOI:** 10.3389/fpsyg.2019.02551

**Published:** 2019-11-15

**Authors:** Jingjing Yang, Yong Zheng

**Affiliations:** ^1^School of Public Health and Management, Chongqing Medical University, Chongqing, China; ^2^Research Center for Medicine and Social Development, Chongqing Medical University, Chongqing, China; ^3^Faculty of Psychology, Southwest University, Chongqing, China

**Keywords:** successes, problems, adult children, parents’ feelings, intergenerational ambivalence, path analysis, health outcomes, Confucian culture

## Abstract

Several studies have focused on adult children’s successes and problems and implications for their own well-being, but few studies have paid attention to their implications for adult children’s health outcomes. In the present study, we tested the links between perceptions of successes, problems, and their own health outcomes, as well as the mediating role of perceptions of parents’ feelings and intergenerational relationships. Adult children (*n* = 314; age 18–59) completed surveys on perceptions of successes (compared with counterparts, speculated how parents rate their successes, and compared with same-gender parent); problems (self’s, father’s, and mother’s); parents’ feelings (positive and negative); intergenerational relationships (intergenerational ambivalence and instrumental solidarity); and health outcomes [subjective well-being (SWB), psychological distress (PD), and self-rated health (SRH)]. Path analysis was conducted, a bootstrapped test was used. Results showed that perceptions of successes compared with counterparts were positively correlated with SWB and SRH; perceptions of successes compared with counterparts and perceptions of successes compared with same-gender parent were positively correlated with SWB and SRH via parents’ positive feelings; perceptions of successes that speculated how parents rate their successes and perceptions of successes compared with same-gender parent were negatively correlated with PD via parents’ negative feelings. Self’s problems were negatively correlated with SWB via direct ambivalence (DA), and were positively correlated with PD via parents’ negative feelings and DA, while mother’s problems were positively correlated with PD via parents’ negative feelings. There were no significant correlations between father’s problems and adult children’s health outcomes. This study underscores the importance of considering perceptions of parents’ feelings and DA in understanding the mechanisms of an individual’s mental health in family systems. This study sheds lights on considering an individual’s health in family systems and cultural contexts.

## Introduction

Extensive research has documented the degree to which adult children’s successes are positively linked with parents’ well-being (e.g., Ryff et al., 1994; Birditt et al., 2010; Fingerman et al., 2012a). Studies have also predominantly focused on the effects of adult children’s problems on their parents’ psychological distress (PD) (Pillemer and Suitor, 1991), psychological well-being (Greenfield and Marks, 2006; Fingerman et al., 2012a; Kalmijn and De Graaf, 2012; Pillemer et al., 2017b), and relational well-being (Greenfield and Marks, 2006), and even coresidence with problematic adult children impacts parental marital quality (Davis et al., 2018). Yet, little research has specifically examined the links between adult children’s successes and problems and their own health outcomes. However, adult children’s successes and problems may be great psychosocial stressors that are correlated with daily well-being, PD, and physical symptoms (Almeida, 2005; Almeida et al., 2005).

More recently, a smaller but growing body of literature has developed that has examined the reverse direction of influence. Research conducted by Bangerter et al. (2016, 2018a, b) has called attention to the detrimental effects of adult children’s problems and parents’ problems on adult children’s mental health. These findings have demonstrated that stressful events, illnesses, and other problems in adult children’s lives are important predictors of depression and other negative mental health outcomes. However, we still know little about more specific mechanisms regarding the implications of adult children’s problems, as well as successes, for adult children themselves in family systems.

Adult children are a significant source of support as well as a strain for mothers and fathers across the entire life span (Birditt et al., 2010; Fingerman et al., 2013). In China, which become an aging society (World Health Organization [WHO], 2015; National Bureau of Statistics of China, 2018), the demand for old-age care for adult children is soaring (Liu and Hu, 2017; Jia and Fan, 2019). Moreover, the Chinese people have always valued family. As the saying goes, “The foundation of the world is at home.” However, there is little research on the function of feelings perceived by adult children in family systems, such as parents’ feelings. Research conducted in 2013 first called attention to parents’ negative emotions in the parent–child tie and underscored the importance of considering the context of parents’ negative emotional experiences in ties to their adult children (Cichy et al., 2013). The present study defines perceptions of parents’ feelings as adult children reporting perceived parents’ positive and negative feelings regarding their successes and problems.

Ambivalence refers to the simultaneous experience of positive and negative sentiments about the same relationship (Luescher and Pillemer, 1998). Middle-aged and older parents report greater ambivalence toward offspring who have not achieved adult milestones (e.g., marriage or job), and older parents report more ambivalence regarding children with problems due to their continuing dependence and the violation of adult status attainment norms (Aldous et al., 1985; Fingerman et al., 2006; Pillemer et al., 2007a, b, 2012). The achievements of adult children can reduce parental evaluations of ambivalence (Kiecolt et al., 2011). Researchers have not explicitly examined whether adult children’s successes and problems help explain their ambivalence toward their relationships with their parents, which affects their physical and mental health.

Adult children with problems provide less care to parents (Cicirelli, 1983), tend to drain their parents’ resources (Aldous et al., 1985), and are their parents’ least preferred support providers (Pillemer and Suitor, 2002). In contrast, successful children may provide more support to parents (Ryff et al., 1994; Fingerman et al., 2009). Adult children may experience stress due to competing desires to deal with their problems (Johnson, 2013) and to support parents in need (e.g., parents with problems).

This article explores the links between children’s successes and problems and their implications for their own health outcomes in family systems in the context of the Confucian culture of modern China. We considering the perceptions of parents’ feelings and intergenerational relationships, such as ambivalence and instrumental solidarity (intergenerational support). We addressed the questions: (1) Are adult children’s perceptions of successes and problems correlated with their own health outcomes? (2) Do perceptions of parents’ feelings and intergenerational relationships play a significant role in the links between perceptions of successes and problems and their own health outcomes?

### Understanding Successes and Problems in Confucian Culture

Filial piety is an important part of Confucianism (Shang and Lu, 2018) and its connotation developed over time. In ancient times, filial piety was the core spirit of family, clan, and country (Qian, 2018). It is useful to maintain the hierarchy order of seniority and to encourage people strive for the honor of family and country. As the ancient saying goes, “Filial piety is the root of virtue.” In the fine traditional Chinese culture, be it in the past or present, many ideas and moral norms have eternal value, such as filial piety to the elderly (Tang and Tang, 2019; Zhai, 2019). Since ancient times, the Chinese people have advocated filial piety and love for their elder. Over the rheological history of several 1000 years, the Chinese nation has encountered numerous difficulties and hardships. The Chinese culture is developing with the times, making dialectical choices, bringing forth the new, discarding negative factors (e.g., the feudal hierarchy and the order of honor and lowliness), and inheriting positive thoughts (e.g., respect and love for the elderly). Therefore, in modern times, filial piety is still viewed as the core moral values of Confucianism and the fine traditional virtues (Lu, 2017; Tu, 2018). It places greater emphasis on family emotional bonds and requires adult children to care for and take care of their parents in return for their upbringing (Zhou, 2017; Bian and Wang, 2018).

On the one hand, Confucian culture requires children to strive for success to make their parents prominent, honorable, and proud. For example, “It is the ultimate goal of filial piety to cultivate oneself, to make achievements, to bring fame to later generations, to make parents prominent and glorious” (Filial Piety, 2014; Kai zong ming yi the first chapter, Qin and Han dynasties). On the other hand, Confucian culture requires children to avoid problems to achieve filial piety: “The body, hair, and skin are given to us by our parents, we must cherish them and take care of them without destroying or hurting them, which is the beginning of filial piety” (Filial Piety, 2014; Kai zong ming yi the first chapter, Qin and Han dynasties). Therefore, the successes and problems of adult Chinese children may be more closely related to their parents’ feelings.

Successes examined in previous studies in Western countries have usually referred to career (and educational) achievement and romantic relationship performance (Ryff et al., 1994; Birditt et al., 2010; Fingerman et al., 2012a; Cichy et al., 2013), but few understand the abundant connotation of successes in the Chinese Confucian cultural context. Firstly, Chinese culture values career and relationship success as much as Western countries. Starting a career and getting married are advocated and their combination is the benchmark for success—namely, “thirtysomething” (The Analects of Confucius, 2016; Spring and Autumn Period and Warring States Period). Secondly, Chinese culture values a family’s harmony and prosperity. As the saying goes, “Jia he wan shi xing”—that is, “Only if you have a harmonious family, everything can go well.” This usually has two meanings. One refers to harmony in the relationships between husband and wife (relationship successes), and the other represents for harmony between parents and children (intergenerational relationships). Moreover, people encourage children to be outstanding and even surpass their parents or predecessors, advocating inheritance, innovation and transcendence, just as Xunzi (2015) (Zhao at the end of the Warring States Period, his book *Exhortation to Learning*) said: “Blue comes from the indigo plant but is bluer than the plant itself.” In a word, the successes of adult children mainly refer to excellence in career, relationships, and cultivating children; more importantly, adult children in China value their successes compared to their counterparts, and they value their parents’ views, and value whether they can exceed their parents.

In the Confucian culture, there are three levels of filial piety. Most fundamentally, children should avoid physical harm or injury to keep parents worrying (e.g., physical problems). Secondly, children should avoid behavior-style problems that will shame their parents. For example, in the *Book of Rites*, Zengzi states, “it is filial piety not to dishonor one’s body or one’s parents” and “Even after the death of their parents, children should be careful not to give their parents a bad reputation” (Dai De, 2018; Han dynasty, *Da Dai Rites*).” Mencius points out that the five kinds of unfilial behaviors that dishonor parents include idleness, gambling, drinking, lust for money, lust for pleasure, and fighting (Mencius., 2019; Zou at the Warring States Period). Thirdly, “There are three unfilial things, and no offspring is the greatest.” This implies two kinds of problems; one is not getting married, and the other is getting married but not having children, which worries parents about family stability and continuity. Therefore, compared with the two types of problems, physical–emotional problems and lifestyle–behavioral problems, in western studies (Greenfield and Marks, 2006; Birditt et al., 2010; Fingerman et al., 2012a; Gilligan et al., 2015b; Pillemer et al., 2017b; Bangerter et al., 2018a, b), the scope of the problems is similar. The classification in western studies is more detailed according to the nature of problems, namely, the controllable and uncontrollable characteristics. There is no systematic classification of problems, but the Confucian culture attaches more importance to the psychological feelings of the parents brought about by their children’s problems.

Additionally, children should provide care for their parents when their parents are old, weak, or ill so that parents can spend their later lives happily. If adult children cannot do this, there are two possibilities. One is that the adult children have problems so that they are unable to do so, and the other is that they are unwilling to support their parents (e.g., disrespect parents). Providing care to parents is a stressful, especially when parents become ill or have other problems (Amirkhanyan and Wolf, 2006; Kim et al., 2008; Timko et al., 2009; Low et al., 2012; Lin et al., 2013; Bangerter et al., 2018a, b), whether or not the children serve as primary caregivers to them. Therefore, both problems of adult children and problems of parents are significant in adult children’s lives.

### Successes and Problems and Health Outcomes

Adult children’s successes and problems are closely related to their parents’ health outcomes. For example, Fingerman et al. (2012a) investigated the problems and successes of multiple grown children and their effects on middle-aged parents’ well-being, and found that having one child with problems predicted poorer parental well-being. Furthermore, the more problems in the family, the worse the parental well-being. Having one successful child did not predict well-being, but having multiple grown children with higher total success in the family predicted enhanced parental well-being. This suggested that middle-aged parents’ well-being is tied to the successes and failures of their adult children and that parents derive benefits and detriments from their adult children turn out. This is exactly what Confucian culture encourages adult children to pay attention to, hoping that children can honor and repay their parents.

Adult children’s successes and problems may be directly related to their own health outcomes. Almeida (2005) proposed a model showing that sociodemographic, psychosocial and health factors are associated with adults’ exposure and reactivity to daily stressors and their daily well-being, and interpersonal tension and network tension were more predictive of PD and physical symptoms than other types of stressors.

Parents’ problems may be included in adult children’s perceptions of intergenerational relationships and can also affect adult children’s physical and mental health. Another study found that mothers’ persistent pain symptoms had no impact on emotional closeness or tension in their relationships with their adult children (Pillemer et al., 2007a), while Bangerter et al. (2018a, b) found that perception of one’s and one’s mother’s problems are relevant to levels of support and stress. Thus, we also considered mothers’ and fathers’ problems in the current study.

### The Potential Mediator: Parents’ Negative and Positive Feelings

The successes and problems of adult children can lead parents to develop corresponding positive or negative emotions. Parents experience emotions associated with unmet goals and future concerns in relationships with less successful children, such as guilt, anger, disappointment, and worry (Cichy et al., 2013). Meanwhile, parents report feelings of pride and joy (Ryff et al., 1994; Nelson et al., 2014) in seeing their grown children excel. Importantly, adult children can experience affective solidarity in intergenerational relationships (Bengtson and Roberts, 1991) and favoritism (Jensen et al., 2017; Suitor et al., 2017; Peng et al., 2018) or disfavoritism from parents (Suitor et al., 2016, 2017, 2018; Peng et al., 2018). Moreover, previous research has indicated that older Korean adults’ well-being and their family caregivers’ well-being can influence one another (Kim et al., 2013). Thus, adult children may perceive their parents’ specific feelings toward them, which can impact their well-being. Therefore, we inferred that adult children’s perceptions of their parents’ feelings may play a mediating role in the associations between perceptions of successes and problems and their own health outcomes.

### The Potential Mediator: Intergenerational Relationships

Successes and problems of adult children are associated with intergenerational relationships. Firstly, parents would report greater ambivalence regarding children with problems and those with less success (Birditt et al., 2010). Secondly, the instrumental solidarity, which is the amount of intergenerational support, varies in the relationship of adult children with different levels of success and problems (Fingerman et al., 2009, 2012b; Cheng et al., 2015; Bangerter et al., 2018a, b).

Intergenerational relationships are linked with health outcomes. Previous research has found that variations in relationship quality accounted for the effect of successful children on parental well-being (Fingerman et al., 2012a, b). Furthermore, parents and offspring who self-reported greater ambivalence showed poorer psychological well-being. Parent reports of ambivalence were associated with poorer physical health (Fingerman et al., 2008).

Intergenerational relationship may be a potential mediator for the effects of successes and problems on health outcomes. Previous research has tested relationship qualities as proxy mediators of the association between children’s problems or successes and parental well-being and has found that the effect of successful children (not the children with problems) on parental well-being maybe mediated by both positive and negative relationship qualities (Fingerman et al., 2012a).

**FIGURE 1:**
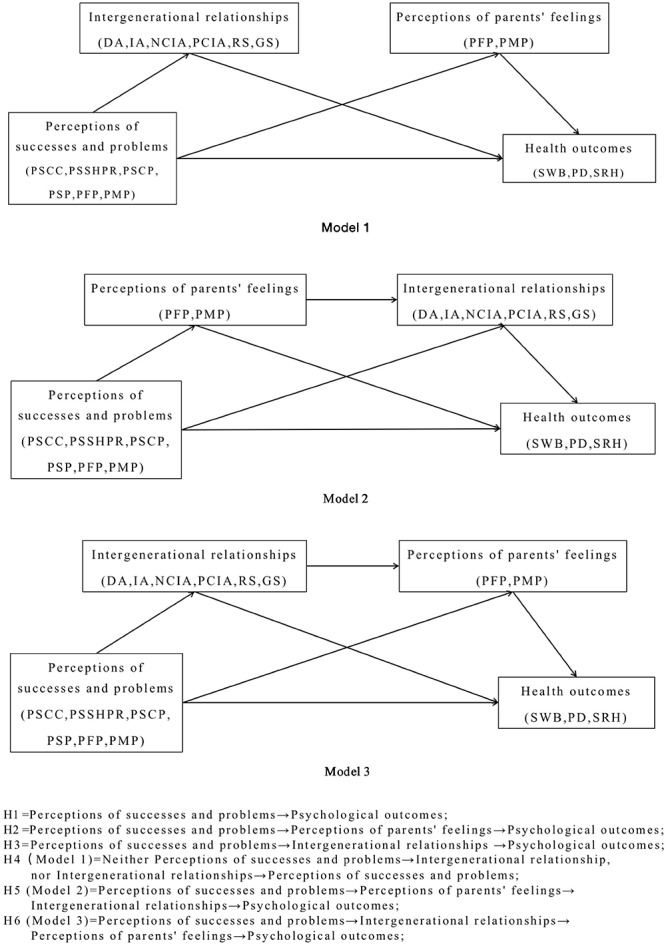
Hypothesized models indicating the associations between perceptions of successes and problems and psychological outcomes and the mediating roles of perceptions of parents’ feelings and intergenerational relationships.

Overall, previous studies have mainly focused on middle-aged or elderly parents’ assessment of adult children’s successes and problems, their perception of intergenerational relationships, and the implications for parents’ physical and mental health, but they have ignored relevant perceptions and the health of the adult children, who are potentially care providers and must be paid sufficient attention. Therefore, this study will address the following questions: (1) Are adult children’s perceived successes and problems directly associated with their own health outcomes? (2) Are adult children’s perceived successes and problems associated with their own health outcomes via their perceptions of their parents’ feelings or intergenerational relationships? We addressed a series of hypotheses as follows (Figure 1).

Hypothesis 1: Perceptions of successes and problems have close relationships with health outcomes, namely the existence of a “perceptions of successes and problems → health outcomes” direct path.

Hypothesis 2: Perceptions of parents’ feelings play a mediating role in the relationship between perceptions of successes and problems and health outcomes, namely the existence of a “perceptions of successes and problems → perceptions of parents’ feelings → health outcomes” path.

Hypothesis 3: Intergenerational relationships plays a mediating role in the relationship perceptions of successes and problems and subjective well-being (SWB) and self-rated health (SRH), namely the existence of a “perceptions of successes and problems → intergenerational relationships → health outcomes” path.

Hypothesis 4 (Model 1): Perceptions of parents’ feelings and intergenerational relationships do not play a serial mediating effect in the model, namely the non-existence of neither a “perceptions of successes and problems → perceptions of parents’ feelings → intergenerational relationships → health outcomes” path or a “perceptions of successes and problems → intergenerational relationships → perceptions of parents’ feelings → health outcomes” path.

Hypothesis 5 (Model 2): Perceptions of successes and problems could significantly predict health outcomes through the serial mediating role of perceptions of parents’ feelings and perceptions of intergenerational relationships, namely the existence of a “perceptions of successes and problems → perceptions of parents’ feelings → intergenerational relationships → health outcomes” path.

Hypothesis 6 (Model 3): Perceptions of successes and problems could significantly predict health outcomes through the serial mediating role of and intergenerational relationships and perceptions of parents’ feelings, namely the existence of a “perceptions of successes and problems → intergenerational relationships → perceptions of parents’ feelings → health outcomes” path.

## Materials and Methods

### Participants

Participants in this study were all adult individuals (age ≥ 18 yeas old) who completed the questionnaires as adult children. They included 317 adult individuals from different families who had at least one living parent. Three of the surveys were excluded from the analysis as the time taken to complete them was too short, and they had incomplete information. Thus, a total of 314 individuals completed the survey and were included in the final study. This study gathered samples from 29 provinces/regions of China, and 45.2% (*n* = 142) was male, and 54.8% (*n* = 172) was female. The mean age was 32.24 years (*SD* = 6.67) and ranged from 19 to 56 years old. One (0.3%) participant reported having a junior high school education, 9 (2.9%) participants having a senior high school education, 273 (86.9%) reported having a college or university education, and 31 (9.9%) reported having a post-graduate education and above. Of the participants, 61 (19.4%) reported being unmarried, 250 (79.6%) reported being married, 3 (1.0%) reported being separated or divorced, and no one reported being widowed. Having no child was reported by 72 (22.9%) individuals, 9 (2.9%) reported being pregnant, 219 (69.7%) reported having only one child, and 14 (4.5%) reported having more than one child.

About the sample size and its sufficiency, I used the software G^∗^Power to calculate the sample size and the statistical power (Faul et al., 2007, 2009; G^∗^Power 3.1 manual, 2017). Test family was set as F tests, and statistical test was linear multiple regression: fixed model, R^2^ diviation from zero. Type of power analysis was using the option “*A priori*: Compute required sample size-given α, power, and effect size.” Input parameters are as followed, α err prob = 0.05, power (1-β err prob) = 0.95, number of tested predictors = 16, total number of predictors = 19. Then, the result of calculation was that total sample size = 217, actual power = 0.95. Therefore, we believe that the sample size of 314 in this study was sufficient to achieve 95% statistical effectiveness.

### Procedure

Only volunteer participants who had at least one living parent were recruited and completed an Internet questionnaire via the professional survey website Wenjuan xing^1^. Respondents were told that the questionnaire was about intergenerational relationships with parents and was entirely anonymous, and they would receive a reward (RMB 5 yuan) for completion of questionnaires. This study was approved by the ethics committees of both Southwest University and Chongqing Medical University of China. All participants gave online informed consent before filling in the questionnaire. Using the independent IP control technique, each participant only took part once and their reports underwent an authenticity check, conducted by the survey company, in terms of their registration information and the time they had spent completing the questionnaire. As adult children, the participants answered questions about themselves and their relationship with their parents. Demographic characteristics of participants in present study are shown in Table 1.

**TABLE 1:** Demographic characteristics of participants in present study.

**Variables**	**Range**	***n* (%)/mean (SD)**
Age	19–56	32.24 (6.67)
Gender	Male	142 (45.2)
	Female	172 (54.8)
Education	Senior high school and below	10 (3.2)
	College or university	273 (86.9)
	Post-graduate and above	31 (9.9)
Marital status	Married	250 (79.6)
	Single/never married	61 (19.4)
	Divorced/separated	3 (1.0)
Family type	None	72 (22.9)
Having children	Pregnant	9 (2.9)
	One	219 (69.7)
	Multiple	14 (4.5)

**N* = 314.*

### Measures

#### Predictors

We accessed perceptions of successes and problems based on the participants’ evaluations.

##### Perceptions of successes

Adult children were asked to rate their own success from three perspectives, namely, perceptions of successes compared to counterparts (PSCC), perceptions of successes speculating on how parents rating (PSSHPR), and perceptions of successes compared to parent (PSCP). The first item was intended to measure how successful they felt compared to their peers, the second was intended to speculate about how successful they were when their parents judged them compared to their peers, and the third was intended to measure how successful they felt compared to their parents of the same sex. Each measure of successes included three aspects: academic achievements or career, marriage or romantic relationships, and parenting or cultivating children. Each aspect was measured by one item. The first two items were derived from previous studies (Ryff et al., 1994; Fingerman et al., 2009), while the last one was self-compiled. Thus, the measure of successes consisted of nine items.

To measure PSCC, for example, the first item was, “how would you evaluate your success in academic, occupational, or career achievement compared to your peers?” The second item was, “How would you evaluate your success in romantic relationships and marriage compared to your peers?” The third item was, “How would you rate your success in parenting, cultivating, and educating your children compared to your peers? (And if you are not under the situation described right now, please imagine and perceive if you do in the future).” PSSHPR and PSCP measures were similar to those of PSCP, but the main questions were, “How do you feel your parents would evaluate your success in. compared to your peers?” and “How would you evaluate your success in. compared to your same-gender parent?”

The scores of the three indicators were differed. PSCC and PSSHPR used a seven-point Likert rating scale, and PSCP used a five-point Likert rating scale. The seven-point Likert rating scale scored here as follows: 1 = not at all successful, 2 = less successful, 3 = somewhat less successful, 4 = about the same as your peers, 5 = somewhat more successful, 6 = more successful, and 7 = much more successful than your peers; the five-point Likert rating scale scored here as follows: 1 = less successful, 2 = somewhat less successful, 3 = about the same as your peers, 4 = somewhat more successful, and 5 = more successful. Then, the answers of PSCP were multiplied by 7 and then divided by 5 for the weights, which calculated the total score. High scores indicated that the adult children perceived high-levels of successes.

For PSCC, Range = 5–21, Mean = 15.72, *SD* = 3.02, α = 0.82; for PSSHPR, Range = 4–21, Mean = 15.97, *SD* = 2.95, α = 0.81; for PSCP, Range = 4.2–21, Mean = 12.75, *SD* = 4.04, α = 0.76. The fit indices used for the evaluation of the CFA model, χ^2^ = 60.78, df = 21, χ^2^/df = 2.89 < 3, CFI = 0.97 > 0.95, TLI = 0.95 > 0.90, RMSEA = 0.08 with 95%CI [0.06–0.10], and SRMR = 0.04 < 0.08 showed that the measurement model of perceptions of successes had a good fit with the data.

##### Perceptions of problems

The measurement of perceptions of problems included three indicators, namely perceptions of self’s problems (PSPs), perceptions of father’s problems (PFPs), and perceptions of mother’s problems (PMPs). The measurements were based on previous studies (Greenfield and Marks, 2006; Birditt et al., 2010; Fingerman et al., 2011, 2012b; Pillemer et al., 2017b; Bangerter et al., 2018a,b) and were developed to include several issues of particular importance in Chinese culture (such as, physical injuries) into the previously controllable and uncontrollable measurement framework. The participants were asked to fill out the checklist to report whether they experienced self, mother, and father problems during the past 2 years. The contents of the checklist were as follows: (1) general physical health problems (such as short time hospitalization for illness, chronic diseases, physical injuries, crime, and childless); (2) serious physical health problems (heavy illness, disability, physical defects, mental retardation, or serious injury); (3) emotional or psychological problems (such as autism and depression); (4) general behavioral problems (such as Internet addiction, misconduct, and alcoholism); (5) serious behavior problems (such as drug addiction, crime, detention, and prison); (6) employment issues (such as unemployment, employment difficulties, and financial difficulties); (7) family problems (such as difficulty choosing a spouse, family disharmony, divorce, and widowhood), (8) other problems; and (9) no problem. Given that our primary concern was the effects of problems experienced by particular individual, rather than the differential effects of particular types of problems, we summed the number of problems. Each adult child received three score of problems. Higher scores indicated more problems.

For PSP, Range = 0–7, Mean = 0.94, *SD* = 1.05, Median = 1.00, Mode = 0, Skewness = 1.37, Kurtosis = 3.16; for PFP, Range = 0–4, Mean = 0.94, *SD* = 0.89, Median = 1.00, Mode = 1, Skewness = 1.05, Kurtosis = 1.15; for PMP, Range = 0–5, Mean = 0.88, *SD* = 0.86, Median = 1.00, Mode = 1, Skewness = 1.16, Kurtosis = 1.99.

#### Potential Mediators

The potential mediating variables to be explored in this study were perceived parents’ negative and positive emotions as well as intergenerational relationships.

##### Perceived parent’s negative and positive feelings

There are two measures of perceived parent’s feelings, namely parent’s negative feelings (PNFs) and parent’s positive feelings (PPFs). Participants were asked to report their perceptions of their parents’ negative and positive feelings according to their own successes and problems during the past year. Negative items used four indicators: guilt, anger, disappointment and worry, which were used in previous studies (Cichy et al., 2013). We added four corresponding positive words that may perceive from parents as happy, joyful, proud (Peters et al., 2006), and satisfied. Examples of items were, “During the past year, about how often did you feel your parents felt guilty for you?” and “During the past year, about how often did you feel your parents were proud of you?” Each item was scored from 1 = “none of the time” to 5 = “all of the time.”

For PNF, high scores indicated that participants perceived a high-level parents’ negative feelings. Range = 4–18, Mean = 8.90, *SD* = 3.30, α = 0.83; for PPF, high scores indicated that participants perceived a high level of parents’ positive feelings. Range = 5–20, Mean = 14.80, *SD* = 2.80, α = 0.83. The fit indices used for the evaluation of the CFA model, χ^2^ = 35.75, df = 17, χ^2^/df = 2.10 < 3, CFI = 0.98 > 0.95, TLI = 0.97 > 0.95, RMSEA = 0.059 < 0.60 with 95%CI [0.03–0.09], and SRMR = 0.04 < 0.08 showed that the measurement model of perceptions of parents’ feelings had a good fit with the data.

##### Intergenerational relationship

Based on the theory of intergenerational ambivalence and the theory of intergenerational solidarity, we choose the indicators of intergenerational ambivalence which best reflect intergenerational emotional complexity and the indicators of instrumental solidarity which can best reflect the intergenerational function.

###### Intergenerational ambivalence

The indicators of intergenerational ambivalence include direct ambivalence (DA), indirect ambivalence (IA), negative component of indirect ambivalence (NCIA), and positive component of indirect ambivalence (PCIA). The measurement of DA used one item to directly ask participants “I feel contradictory emotions/mixed emotions toward him/her (the target parent)”(cf. Suitor et al., 2011). The measurement of IA was firstly assessed with negative and positive components separately. Three items were used to measure the NCIA, namely, “I feel nervous with him/her.” “She will criticize and accuse me, making me feel uncomfortable and unpleasant.” “He/she has too much need and requirement for me. “Three items were used to measure PCIA, namely, “I and he/she is very close”; “I can feel his/her love and concern for me”; “I am very happy with her” (cf. Fingerman et al., 2006, 2008; Willson et al., 2006; Lendon et al., 2014; Gilligan et al., 2015a). All items were scored from 1 = never to 5 = very often. Secondly, the Griffin algorithm (Gilligan et al., 2015a) was used to calculate the value of IA, indirect ambivalence (IA) = (positive + negative)/2 – — positive – negative — + 1.5.

For DA, high scores indicated a perceived high level of direct intergenerational ambivalence. Range = 1–5, Mean = 2.27, *SD* = 1.09. For IA, high scores indicated a perceived high level of indirect intergenerational ambivalence. Range = 0.5–6, Mean = 2.55, *SD* = 1.25. For NCIA, high scores indicated negative intergenerational relationship quality. Range = 1–5, Mean = 3.93, *SD* = 0.93, α = 0.86. For PCIA, high scores indicated positive intergenerational relationship quality. Range = 1–5, Mean = 2.16, *SD* = 0.89, α = 0.77.

###### Instrumental solidarity

According to Bengtson’s theory of intergenerational solidarity (Bengtson and Roberts, 1991), instrumental solidarity refers to mutual support and help between adult children and middle-aged and elderly parents. The Intergenerational Support Scale (ISS, Fingerman et al., 2009) was used to measure participants’ receiving support (RS) and giving support (GS) within their intergenerational relationships. The ISS measured six types of support, including companionship, talking about daily events, giving emotional support, giving practical help, giving advice, and giving financial support (e.g., “How often do your parents provide you with emotional support?” or “How often do you provide your parents with emotional support?”). All items were scored from 1 = never/rarely to 5 = always. High scores indicated a high level of support giving or receiving. For RS, Range = 6–30, Mean = 21.72, *SD* = 4.67, α = 0.84; for GS, Range = 7–30, Mean = 21.43, *SD* = 4.47, α = 0.84.

#### Outcome Variables

Health outcomes were measured from both psychological and physical two aspects. Three indicators were selected, namely SWB, PD, and SRH. We measured both SWB and PD as indicators of psychological outcomes because the World Health Organization (WHO) recommends that positive and negative feelings are key components of psychological functioning (The WHOQOL Group, 1998; Fingerman et al., 2008). Furthermore, SRH was used as an indicator of physical health. Because SRH is a valid and reliable measure of general physical health (Bombak, 2013), even the correlation with mortality is better than that of objective health indicators (Mossey and Shapiro, 1982; Idler and Benyamini, 1997; Benyamini and Idler, 1999).

##### Subjective well-being

Six-item measure was used to assess positive psychological outcomes. It was composed of an overall rating of a life-satisfaction item and five positive affect items based on the research of Diener and Emmons (1984) and Newsom et al. (2005). Participants answered the life satisfaction question “All things considered, how satisfied are you with your life these days?” on an adapted scale ranging from 0 (very unsatisfied) to 10 (very satisfied). The remaining items asked the participants to rate the extent to which five adjectives (happy, joyful, pleased, enjoying myself, and satisfied) described their feelings over the past month, with ratings made on a scale ranging from 0 (very slightly or not at all) to 10 (very much). High scores indicated a high level of SWB. Range = 4–60, Mean = 39.61, *SD* = 11.93, α = 0.96. Checking the unidimensional structure of this questionnaire was performed by a CFA and the indices used for the evaluation of the CFA model, χ^2^ = 19.53, df = 8, χ^2^/df = 2.44 < 3, CFI = 0.99 > 0.95, TLI = 0.99 > 0.95, RMSEA = 0.068 with 95%CI [0.03–0.11], and SRMR = 0.01 < 0.08 showed that the measurement model of SWB had a good fit with the data.

##### Psychological distress

The Kessler Psychological Distress Scale (K10), a measure of PD, was used to assess negative psychological outcomes, and it is suitable for mental health measurement (Andrews and Slade, 2001). Participants were asked to report the extent to which they had experienced certain items during the previous 4 weeks. An example item was, “During the past 4 weeks, about how often did you feel tired out for no good reason?” Each item was scored from 1 = “none of the time” to 5 = “all of the time.” Scores of the 10 items were then summed. Low scores indicated low levels of PD and high scores indicated high levels of PD. Range = 10–45, Mean = 19.64, *SD* = 7.75. Cronbach’s alpha for these items in the present study was α = 0.95.

##### Self-rated health

Self-rated health was used as an overall perception of physical health status. SRH was measured by asking participants, “Would you rate your physical health as very good, good, fair, poor or very poor?” Respondents were assigned scores of 5, 4, 3, 2, or 1, respectively. Higher scores indicated better SRH. In the present study, Range = 2–5, Mean = 4.09, *SD* = 0.57.

#### Demographic Characteristics

The participants’ demographic characteristics, which were statistically controlled in the mediation models. These included gender, age, education, marital status, and family type. Gender was coded 1 = male and 0 = female. Age was calculated by “survey date – date of birth” and rounded in years. Education was coded as 1 = senior high school and below, 2 = college education, and 3 = post-graduate education or higher. Marital status was coded as 1 = separated or divorced, 2 = unmarried, 3 = married. Family type was coded as 1 = having no child, 2 = pregnant, 3 = having only one child, and 4 = having more than one child.

### Statistical Analysis

Descriptive data analysis (e.g., mean and SD) was performed using SPSS 23 (IBM, Armonk, NY, United States). Cronbach’s alpha coefficient was calculated to test the reliability of each scale. The psychometric characteristics of the questionnaires of perceptions of successes, parents’ feelings and SWB were checked through a CFA (method of estimation was maximum likelihood) performed by Mplus 7.4 (Muthén and Muthén, 1998-2012). Statistical significance was set at *p* < 0.05.

The hypothesized model had two exogenous variables (perceptions of successes and perceptions of problems) and three endogenous variables (parents’ feelings, intergenerational relationships, and psychological outcomes). All variables are labeled with their respective indicators in parentheses (see Figure 1). Covariates (gender, age, education, marital status, and family type), were also entered each analysis. The hypothesized pathways were tested using the maximum likelihood estimation (ML) method to test the hypotheses and detect any significant pathways (*p* < 0.05) in the three models for SWB, PD and SRH, separately.

The hypothesized models (Figure 1) were tested by Mplus 7.4 using the path analysis method and syntax commands including MODEL INDIRECT (Muthén and Muthén, 1998-2012) and received the model fit information. Loglilikelihood (LL), Akaike information criterion (AIC), Bayesian information criterion (BIC), and Adjusted BIC were used to compare the models. The final pathway models were presented in significant pathways, standardized regression weights (β), and *p*-values. Direct and indirect paths related to perceptions of successes and problems are further discussed in the following section.

The BOOTSTRAP = 1000 option was used in conjunction with the standardized CINTERVAL (BCBOOTSTRAP) option to obtain indirect effects of bootstrapped standard errors and bootstrap confidence intervals. Empirical 95% confidence intervals (CI) did not have zero, indicating that the indirect effect was statistically significant.

## Results

### Common Method Bias Test

After completion of the data collection, the Harman’s single-factor test was used to test for common method bias. The results showed that the first factor only accounted for 27.60% of variance, which was much lower than the critical standard of 40%, suggesting that common method bias was not obvious.

### Results of Tested Hypothesized Model

The model fit information is listed in Table 2. We compared the model fit information and found that Model 2 (see also, Figure 1 Model 2) was best in AIC, BIC, Adjusted BIC, and LL in terms of predicting SWB, PD, and SRH, with the minimum value of all three models. This indicates that Model 2 fit the data best.

**TABLE 2:** Model fit information of multiple mediation effects.

**Outcome variable**	**Criteria**	**AIC**	**BIC**	**Adjusted BIC**	***LL***	***df***	***p***
SWB	Model 1	14933.52	15248.47	14982.05	1111.22	59	0.000
	Model 2	14795.71	15155.65	14851.17	949.40	47	0.000
	Model 3	14844.40	15204.34	14899.86	998.10	47	0.000
PD	Model 1	14891.94	15206.89	14940.47	1069.63	59	0.000
	Model 2	14754.13	15114.07	14809.59	907.82	47	0.000
	Model 3	14802.82	15162.76	14858.28	956.51	47	0.000
SRH	Model 1	15086.35	15401.30	15134.87	1264.04	59	0.000
	Model 2	14948.54	15308.48	15004.00	1102.23	47	0.000
	Model 3	14997.23	15357.17	15052.69	1150.92	47	0.000

**N* = 314. SWB, subjective well-being; PD, psychological distress; SRH, self-rated health. AIC, Akaike information criterion; BIC, Bayesian information criterion; Adjusted BIC, sample-size adjusted Bayesian information criterion; LL, loglikelihood.*

**FIGURE 2:**
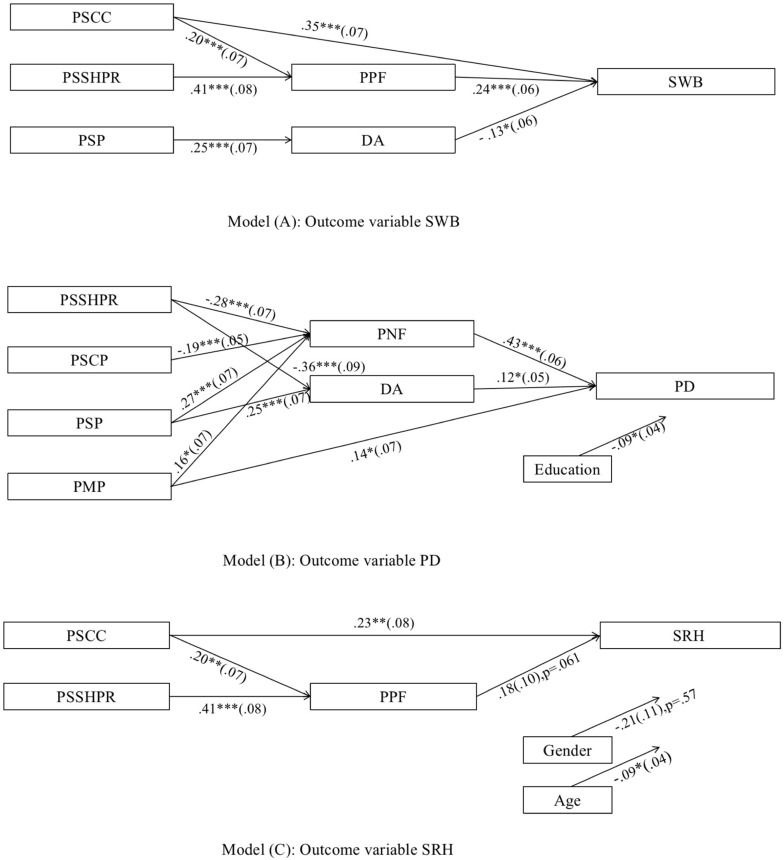
Path models depicting effects of perceptions of successes and problems on health outcomes mediated through parents’ feelings and direct ambivalence, controlling for gender, age, education, marital status, and family type. Standardized path coefficients (β) are reported. Not shown are non-significant pathways. Variables and significant pathways not related to the associations between perceptions of successes and problems and health outcomes are also omitted for ease of viewing. ^∗^*p* < 0.05, ^∗∗^*p* < 0.01, ^∗∗∗^*p* < 0.001.

The specific path analysis of the main effects is shown in Figure 2 and Table 3 (see Supplementary Table 1 for more information) The standardized coefficients embedded in the path models indicated that PSCC had a statistically significant, direct association with SWB (β = 0.35, *SE* = 0.07, 95%CI[0.21, 0.48], *p* < 0.001) and SRH (β = 0.23, *SE* = 0.08, 95%CI[0.08, 0.39], *p* < 0.01); PMP had statistically significant, direct associations with PD (β = 0.14, *SE* = 0.07, 95%CI[0.02, 0.29], *p* < 0.05). Therefore, Hypothesis 1 is supported.

**TABLE 3:** The Significant direct, indirect and specific indirect effects of perceptions of successes and problems on health outcomes.

**Model pathways**	**Estimate**	***SE***	**95% CI**	**Hypothesis tested**
**Model (A): outcome variable SWB**				
**Direct effects**				
PSCC→ SWB	0.35^∗∗∗^	0.07	[0.21, 0.48]	Support H1
**Indirect effects**				
PSCC→ SWB	0.07^∗^	0.04	[0.01, 0.14]	
PSSHPR→ SWB	0.16^∗∗∗^	0.04	[0.09, 0.26]	
**Specific indirect effects**				
PSCC→ PPF → SWB	0.05^∗^	0.02	[0.01, 0.10]	Support H2
PSSHPR→ PPF → SWB	–0.10^∗∗^	0.03	[0.04, 0.18]	Support H2
PSP→ DA → SWB	−0.03^+^	0.02	[−0.08, −0.01]	Support H3
**Model (B): outcome variable PD**				
**Direct effects**				
PMP →PD	0.14^∗^	0.07	[0.02, 0.29]	Support H1
**Indirect effects**				
PSSHPR→PD	–0.22^∗∗∗^	0.05	[−0.31, −0.13]	
PSCP→ PD	–0.10^∗∗^	0.03	[−0.17, −0.04]	
PSP→ PD	0.21^∗∗∗^	0.05	[0.12, 0.32]	
PMP→ PD	0.11^∗^	0.05	[0.02, 0.22]	
**Specific indirect effects**				
PSSHPR → PNF → PD	–0.12^∗∗∗^	0.04	[−0.20, −0.06]	Support H2
PSCP→ PNF → PD	–0.08^∗∗^	0.03	[−0.14, −0.04]	Support H2
PSP→ PNF → PD	0.11^∗∗∗^	0.04	[0.06, 0.20]	Support H2
PMP→ PNF → PD	0.07^∗^	0.03	[0.02, 0.15]	Support H2
PSSHPR → DA → PD	−0.04^+^	0.02	[−0.09, −0.01]	Support H3
PSP→ DA → PD	0.03^+^	0.02	[0.004, 0.07]	Support H3
**Model (C): outcome variable SRH**				
**Direct effects**				
PSCC→ SRH	0.23^∗∗^	0.08	[0.08, 0.39]	Support H1
**Indirect effects**				
PSCC→ SRH	0.05	0.06	[−0.008, 0.03]	
**Specific indirect effects**				
PSCC→ PPF → SRH	0.07^+^	0.04	[0.08, 0.39]	Support H2

**N* = 314. CI, confidence interval; SWB, subjective well-being; PSCC, perceptions of successes compared to counterparts; PSSHPR, perceptions of successes speculating on how parents rating; PPF, parent’s positive feelings; PSP, perceptions of self’s problems; DA, direct ambivalence; PD, psychological distress; PMP, perceptions of mother’s problems; PSCP, perceptions of successes compared to parent; PNF, parent’s negative feelings; SRH, The path coefficient in the model is standardized coefficient. ^+^*p* < 0.1, ^∗^*p* < 0.05, ^∗∗^*p* < 0.01, ^∗∗∗^*p* < 0.001.*

Both PNF and PPF mediated the association between perceptions of successes and problems and health outcomes, and the significant mediating roles were: (1) the mediating role of PPF for SWB was revealed in the indirect path from PSCC to SWB via PPF(β = 0.05, *SE* = 0.02, 95%CI[0.01, 0.10], *p* < 0.05), and the indirect path from PSSHPR to SWB via PPF(β = −0.10, *SE* = 0.03, 95%CI[0.04, 0.18], *p* < 0.01); (2) the mediating role of PNF for PD was revealed in the indirect path from PSSHPR to PD via PNF(β = −0.12, *SE* = 0.04, 95%CI[−0.20, −0.06], *p* < 0.001), the indirect path from PSCP to PD via PNF(β = −0.08, *SE* = 0.03, 95%CI[−0.14, −0.04], *p* < 0.01), the indirect path from PSP to PD via PNF(β = 0.11, *SE* = 0.04, 95%CI[0.06, 0.20], *p* < 0.001), and the indirect path from PMP to PD via PNF(β = 0.07, *SE* = 0.03, 95%CI[0.02, 0.15], *p* < 0.05); and (3) the mediating role of PPF for SRH was revealed in the indirect path from PSCC to SRH via PPF(β = 0.07, *SE* = 0.04, 95%CI[0.08, 0.39], *p* < 0.1). Therefore, Hypothesis 2 is supported.

Only DA mediated the association between perceptions of successes and problems and health outcomes, and the significant mediating roles were: (1) the mediating role of DA for SWB was revealed in the indirect path from PSP to SWB via DA (β = −0.03, *SE* = 0.02, 95%CI[−0.08, −0.01], *p* < 0.1); and (2) the mediating role of DA for PD was revealed in the indirect path from PSSHPR to PD via DA(β = −0.04, *SE* = 0.02, 95%CI[−0.09, −0.01], *p* < 0.1), and the indirect path from PSP to PD via DA (β = 0.03, *SE* = 0.02, 95%CI[0.004, 0.07], *p* < 0.1). Therefore, hypotheses 3 is supported.

The serial mediating roles of parents’ feelings and intergenerational relationships in associations between perceptions of successes and problems and health outcomes were not significant in the models. Therefore, Hypothesis 4 is not supported.

**FIGURE 3:**
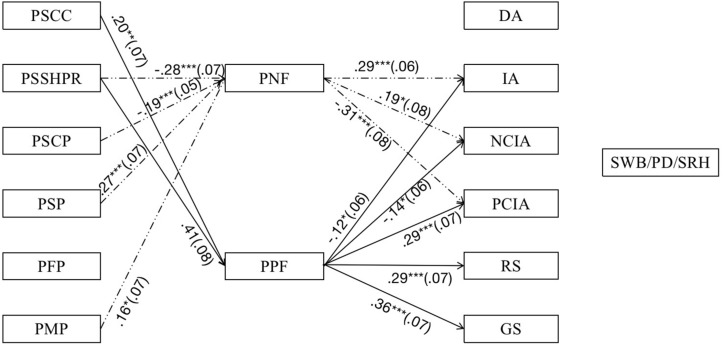
Path models depicting effects of perceptions of successes and problems on intergenerational relationships mediated through parents’ feelings, controlling for gender, age, education, marital status, and family type. Standardized path coefficients (β) are reported. Solid lines refer to pathways through PPF, and dashed lines refer to pathways though PNF. Non-significant pathways, significant direct pathways from perceptions of successes and problems to intergenerational relationships, and pathways shown in Figure 1 are omitted for ease of viewing. ^∗^*p* < 0.05, ^∗∗^*p* < 0.01, ^∗∗∗^*p* < 0.001.

Although Hypothesis 4 is not supported, however, the specific indirect pathways between perceptions of successes and problems and intergenerational relationships are shown in Figure 3 and Table 4 (see Supplementary Table 2 for more information) Perceptions of successes and problems (apart from PFP) had significant associations with intergenerational relationships (apart from DA) mediated by both PNF and PPF. The significant mediating roles were: (1) the mediating role of PNF for intergenerational relationships revealed in indirect paths from PSSHPR, PSCP, PSP, and PMP to IA, NCIA, and PCIA, separately; and (2) the mediating role of PPF for intergenerational relationships revealed in indirect paths from PSCC and PSSHPR to IA, NCIA, PCIA, RS, and GS, separately.

**TABLE 4:** The specific indirect effects for perceptions of successes and problems on intergenerational relationships and parents’ feelings.

**Model pathways**	**Estimate**	***SE***	**95% CI**
PSCC → PPF → IA	–0.03	0.02	[−0.07, −0.004]
PSCC → PPF → NCIA	–0.03	0.02	[−0.07, −0.01]
PSCC → PPF → PCIA	0.06^∗^	0.02	[0.02, 0.11]
PSCC → PPF → RS	0.06^∗^	0.02	[0.02, 0.11]
PSCC → PPF → GS	0.07^∗^	0.03	[0.02, 0.14]
PSSHPR → PNF → IA	–0.08^∗∗∗^	0.02	[−0.14, −0.04]
PSSHPR → PNF → NCIA	−0.05^∗^	0.02	[−0.11, −0.02]
PSSHPR → PNF → PCIA	0.08^∗∗^	0.03	[0.04, 0.16]
PSSHPR → PPF → IA	−0.05^∗^	0.02	[−0.11, −0.01]
PSSHPR → PPF → NCIA	−0.06^∗^	0.03	[−0.12, −0.01]
PSSHPR → PPF → PCIA	0.12^∗∗^	0.04	[0.06, 0.21]
PSSHPR → PPF → RS	0.12^∗∗^	0.04	[0.05, 0.22]
PSSHPR → PPF → GS	0.15^∗∗∗^	0.04	[0.08, 0.24]
PSCP → PNF → IA	–0.05^∗∗^	0.02	[−0.10, −0.02]
PSCP → PNF → NCIA	−0.04^∗^	0.02	[−0.08, −0.01]
PSCP → PNF → PCIA	0.06^∗^	0.03	[0.02, 0.12]
PSP → PNF → IA	0.08^∗∗^	0.03	[0.03, 0.14]
PSP → PNF → NCIA	0.05^∗^	0.03	[0.01, 0.12]
PSP → PNF → PCIA	–0.08^∗∗^	0.03	[−0.15, −0.03]
PMP → PNF → IA	0.05	0.03	[0.01, 0.11]
PMP → PNF → NCIA	0.03	0.02	[0.004, 0.09]
PMP → PNF → PCIA	–0.05	0.03	[−0.11, −0.01]

**N* = 314. CI, confidence interval; PSCC, perceptions of successes compared to counterparts; PPF, parent’s positive feelings; IA, indirect ambivalence; NCIA, negative component of indirect ambivalence; PCIA, positive component of indirect ambivalence; RS, receiving support; GS, giving support; PNF, parent’s negative feelings; PSSHPR, perceptions of successes speculating on how parents rating; PSCP, perceptions of successes compared to parent; PSP, perceptions of self’s problems; PMP, perceptions of mother’s problems. The path coefficient in the model is standardized coefficient. ^∗^*p* < 0.05, ^∗∗^*p* < 0.01, *^∗∗∗^p* < 0.001.*

Surprisingly, as shown in Figures 2, 3, fathers’ problems were not significantly associated with parents’ feelings, intergenerational relationships, or health outcomes.

## Discussion

We examined the links between adult children’s perceptions of successes and problems, parents’ feelings (positive and negative), intergenerational relationships (intergenerational ambivalence and instrumental solidarity), and reports of health outcomes (SWB, PD, and SRH). This research extends our knowledge of the associations between adult Chinese children’s perceptions of successes and problems and their own health outcomes, including discovering the mediating role of perceptions of parents’ positive and negative feelings and DA.

The direct associations between perceptions of successes and problems and health outcomes are significant. Perceptions of successes compared with counterparts had direct associations with SWB and SRH, while perceptions of mothers’ problems were directly associated with PD. This may suggest that successes compared with counterparts are positively correlated with positive health outcomes, while mothers’ problems had positive correlations with negative health outcomes of adult children. The results were consistent with previous research on perceptions of successes compared with counterparts such as persons of the same age or siblings (Birditt et al., 2010) and studies on mothers’ problems related to adult children’s health (e.g., Bangerter et al., 2016). Previous researchers have proposed an interpersonal social-cognitive theory of the self and personality, the relational self, in which knowledge about the self is linked with knowledge about significant others, and each linkage embodies a self–other relationship (Andersen and Chen, 2002). Moreover, studies have used functional MRI experimental designs to explore whether Chinese culture influences the perspective of self-concept (collectivist self contains maternal elements) and have found that the role of the mother in Chinese memory is similar to that of the self, and it is suggested that the Chinese self-schema may include the mother (Zhang, 2005; Zhang et al., 2005). Additionally, researchers have used conservation of resources (CORs) theory to explain this phenomenon (Bangerter et al., 2016), which may help explain how adults utilize various resources to manage stressful circumstances in the family. Both problems of self and mother are stressful events, and successes may represent the volume of resources available to deal with such problems. Yet, we can see that perceptions of parents’ positive feelings can mediated the associations between perceptions of successes and receiving and GS with parents (Figure 3). This suggests that offspring can use personal cognitive resources to care for their parents. Therefore, this study provides a unique extension of COR theory.

Several significant indirect associations exist between perceptions of successes and problems and health outcomes. Perceptions of parents’ positive feelings played a mediating role in the pathways from perceptions of successes (compared with counterparts and speculation on how parents rate their successes) to positive health outcomes (SWB and SRH); perceptions of parents’ negative feelings played a the mediating role in the pathways from perceptions of successes (speculation on how parents rate their successes and compared with same-gender parent) and problems (self’s and mother’s) to the negative health outcome (PD); DA played a mediating role in the pathways from perceptions of successes (speculation on how parents rate their successes) and problems (self and mothers’) to psychological health outcomes (SWB and PD). This may suggest that perceptions of parents’ positive feelings are benefits for both psychological and physical positive health outcomes, and perceptions of parents’ negative feelings are detrimental to adult children’s negative psychological outcomes. This confirmed that adult Chinese children care deeply about parental psychological feelings which brought about by their personal successes and problems. Moreover, DA (nor IA, components of ambivalence, and instrumental solidarity) has complex and mixed implications for psychological outcomes that are both positive and negative. This may have something to do with Chinese people’s emotional reserve and lack of expressiveness. Chinese people experienced more mixed feelings than Westerners and want to feel positive relative to negative emotions (Sims et al., 2015). This also suggests that the DA indicator is recommended to be used in health-related studies as an effective predictor of health variables in the Chinese context.

Through the significant direct and indirect pathways, we could find three ways that perceptions of successes (compared with counterparts, speculation on how parents rate their successes, and compared with same-gender parent) had different mechanisms related with health outcomes. These suggested that different ways of perceiving of successes could shift the level of health outcomes. Previous research has found that parents who saw their children as better adjusted than themselves had lower well-being (Ryff et al., 1994). In the current study, adult children who saw their successes as exceeding those of their same-gender parent had lower PD. Therefore, personal comparisons were significant, negative indicators (Ryff et al., 1994) for parents in Westerners but were significant, positive indicators for adult children among Chinese.

Strangely, fathers’ problems were inactive in the direct or indirect pathways to health outcomes or intergenerational relationships. This result contradicts common sense and may be due to fathers’ stubbornness (Heid et al., 2015, 2016a,b, 2017, 2018). Since fathers tend to be stubborn, it is necessary to ponder and reflect on the reason extra attention is not paid to fathers either emotionally or behaviorally. As fathers cannot express their own sentiment well, they are a potentially vulnerable group to whom sufficient attention must be paid. Perhaps, their stubborn character may comprise a breakthrough for involving personality in intergenerational relationships and understanding the reason fathers receive the cold shoulder. Maybe the reason is that fathers used to be the authority in the family and are now losing their high-ranking status and becoming useless, even with problems. There may, of course, be a huge drop for fathers, and stubbornness may be a defense mechanism, a “reverse” to assert authority and power, resulting in fathers receiving no pity or affective care. However, research on fathers in intergenerational relationships is still limited and needs more attention to explore further mechanisms.

Furthermore, we found that perceptions of parents’ feelings also had mediating roles in the associations between perceptions of successes and problems and intergenerational relationships (apart from DA). Thus, this may suggest that perceptions of parents’ feelings could be used to improve intergenerational relationships (apart from DA).

## Conclusion

Culture is an important force for the survival and development of a nation. Great thinkers, such as Confucius, have expressed ideas and principles that have shaped different cultural traditions and influenced human life. Taking into consideration adult Chinese children, for example, this study found that adult Chinese children’s perceptions of successes and problems had significant implications for their own health outcomes, in which perceptions of parents’ feelings and DA played mediating roles. The results suggest that adult Chinese children are under the influences of Confucian culture, attach importance to their parents’ psychological feelings about their own successes and problems, and consider personal successes and problems by putting them into the family system. This study sheds lights on the consideration of an individual’s health in family systems and cultural contexts.

The current research has a number of limitations. Firstly, although it utilized intergenerational relationships, using indicators to include emotional and instrumental functional aspects—i.e., ambivalence and support—nevertheless, we only investigated the reports of adult children. Previous researchers (Bangerter et al., 2016, 2018b) have explored mothers’ and adult children’s perceptions of support provision during problems or crises and have found distinctions between mother–son and mother–daughter dyads. Thus, using dyadic data and actor partner interaction model (APIM) analysis may help identify distinctions between generations and may explain some inexplicable and obscure results. Moreover, previous studies (Neupert and Carr, 2017; Lin and Wu, 2018) have powerfully demonstrated the existence of a report bias on the part of either parents or children. Therefore, the reports of multiple generations should be considered in future research.

Secondly, this study used horizontal data, so interpretation should be done with caution. A diary approach could be used to assess participants’ reports over time, and to ascertain immediate or delayed effects (Birditt et al., 2016; Bangerter et al., 2018a). Bangerter’s series of studies investigated the provision of help to parents with problems and found that it had significant effects on the circadian cortisol and daily mood of middle-aged children (for four consecutive days). The biological, psychosocial approach, as a comprehensive application, may provide a clearer discussion on the mechanism of action, which is crucial to the formulation and implementation of intervention measures. Therefore, using biological methods is well worth considering in future research.

Thirdly, intergenerational relationships and their effects on adult children’s and older parents’ health and well-being may be usefully explored in more detail. Previous research has illustrated that mothers’ chronic pain has no effect on their relationships with their children, either in terms of intimacy or tension (Pillemer et al., 2017a). Pillemer et al. (2017a) argue that mechanisms may exist that protect adult child caregivers from the stressors that result from a relative’s chronic pain. Whether or not the stress relief mechanism for children exists, it is evident that the typology of the problem matters. We should, therefore, ascertain what kinds of problems or diseases place more pressure on children and their mothers. The fastigial hours may emerge when a mother’s problem is first discovered, and it is worth examining whether the duration of the problem has different psychological effects. If duration does greatly influence adult children’s physical and mental health, in order to ensure better support is given to parents, we should identify and capture the targeted intervention time and propose measures to redress the effects. Therefore, the results of our study have important practical significance. For example, hospitals may provide outpatient services that provide care and love to patients’ families and offer emotional or mental assistance to them.

Overall, future research should consider family and cultural contexts in studying an individual’s physical and mental health, as well as the dyadic data analyzed by APIM. It is better to combine biological with psychosocial approaches and to apply a diary approach to explore the dynamic changes of health outcomes during the stressful issues that emerge and develop.

## Data Availability Statement

The datasets generated for this study are available on request to the corresponding author.

## Ethics Statement

All study participants provided informed consent before they take part in the survey, and the study design was approved by the Chongqing Medical University Ethics Review Board.

## Author Contributions

JY was responsible for the writing of the research plans, data collection and analysis, and thesis writing. YZ was responsible for the guidance of the research plan, supervision of the research implementation, and revision of the manuscript.

## Conflict of Interest

The authors declare that the research was conducted in the absence of any commercial or financial relationships that could be construed as a potential conflict of interest.

## Footnotes

1 https://www.wjx.cn/
