# Supplementary material for: Links Between Perceptions of Successes, Problems and Health Outcomes Among Adult Chinese Children: The Mediating Role of Perceptions of Parents’ Feelings and Intergenerational Relationships
Source: Front Psychol. 2019 Nov 15;10:2551. doi: 10.3389/fpsyg.2019.02551 (PMC6872502; doi:10.3389/fpsyg.2019.02551)
Supplement: Supplementary file 1 [file Table_1.DOCX]

Supplementary Table 1. The direct and indirect effects of predictors and mediators on the health outcomes and 95% confidence intervals (CI).

| **Model pathways** | Model(A):Outcome variable SWB | | |  | Model(B):Outcome variable PD | |  | Model(C):Outcome variable SRH | |
| --- | --- | --- | --- | --- | --- | --- | --- | --- | --- |
|  | **Estimate (SE)** | | **95%CI** |  | **Estimate (SE)** | **95%CI** |  | **Estimate (SE)** | **95%CI** |
| **Direct effects** | | |  |  |  |  |  |  |  |
| **Predictors:** | | |  |  |  |  |  |  |  |
| PSCC | | **.35*** (.07)** | [.21, .48] |  | -.07 (.08) | [-.23, .08] |  | **.23** (.08)** | [.08, .39] |
| PSSHPR | | .04 (.08) | [-.13, .17] |  | 0.04 (.08) | [-.11, .20] |  | -.06 (.09) | [-.25, .10] |
| PSCP | | .05 (.05) | [-.04, .14] |  | -0.08 (.05) | [-.17, .01] |  | -0.07 (.06) | [-.18, .05] |
| PSP | | -.12 (.07) | [-.27, .02] |  | 0.06 (.07) | [-.10, .19] |  | -0.14 (.08) | [-.30, .02] |
| PFP | | -.01 (.08) | [-.16, .14] |  | 0.03 (.07) | [-.10, .19] |  | 0.12 (.10) | [-.06, .32] |
| PMP | | -.03 (.07) | [-.16, .11] |  | **0.14* (.07)** | [.02, .29] |  | -0.13 (.09) | [-.29, .06] |
| **Potential mediators:** | | |  |  |  |  |  |  |  |
| DA | | **-.13* (.06)** | [-.26, -.02] |  | **0.12* (.05)** | [.01, .22] |  | -0.07 (.07) | [-.22, .05] |
| IA | | -.12 (.09) | [-.28, .06] |  | 0.09 (.10) | [-.14, .25] |  | 0.15 (.12) | [.11, .36] |
| NCIA | | .11 (.08) | [-.04, .27] |  | 0.06 (.11) | [-.11, .30] |  | 0.06 (.11) | [-.13, .30] |
| PCIA | | -.06 (.05) | [-.15, .05] |  | -0.09 (.06) | [-.20, .02] |  | 0.004 (.06) | [-.12, .12] |
| RS | | -.03 (.07) | [-.17, .10] |  | 0.01 (.06) | [-.11, .13] |  | 0.06 (.08) | [-.10, .22] |
| GS | | .11 (.07) | [-.03, .26] |  | 0.01 (.06) | [-.12, .13] |  | -0.04 (.08) | [-.19, .13] |
| PNF | | -.07 (.06) | [-.20, .05] |  | **0.43*** (.06)** | [.31, .55] |  | -0.07 (.07) | [-.22, .07] |
| PPF | | **.24***(.06)** | [.11, .35] |  | 0.04 (.06) | [-.08, .16] |  | 0.18 (.10) | [-.01, .37] |
| **Control variables:** | | |  |  |  |  |  |  |  |
| Gender | | .07 (.04) | [-.01, .16] |  | -0.004 (.04) | [-.08, .07] |  | **0.11* (.06)** | [.01, .22] |
| Age | | -.04 (.05) | [-.15, .06] |  | -0.06 (.06) | [-.17, .04] |  | **-0.26***(.07)** | [-.40, -.14] |
| Education | | .01 (.04) | [-.08, .09] |  | **-0.09* (.04)** | [-.17, -.01] |  | -0.05 (.06) | [-.16, .06] |
| Marital status | | .03 (.09) | [-.15, .19] |  | -0.06 (.07) | [-.20, .08] |  | 0.01 (.11) | [-.21, .21] |
| Family style | | -.13 (.09) | [-.30, .03] |  | 0.14 (.08) | [.002, .31] |  | -0.07 (.12) | [-.31, .15] |
| **Indirect effects** | | |  |  |  |  |  |  |  |
| PSCC | | **.07*(.04)** | [.01, .14] |  | .03 (.05) | [-.06, .12] |  | .07(.04) | [-.002, .02] |
| PSSHPR | | **.16***(.04)** | [.09, .26] |  | **-.22*** (.05)** | [-.31, -.13] |  | .05(.06) | [-.02,.03] |
| PSCP | | .003(.02) | [-.04, .05] |  | **-.10** (.03)** | [-.17, -.04] |  | -.01(.03) | [-.01, .04] |
| PSP | | -.06(.04) | [-.14,.002] |  | **.21*** (.05)** | [.12, .32] |  | .04(.04) | [-.04, .05] |
| PFP | | .03(.02) | [-.04, .10] |  | -.02 (.05) | [-.13, .07] |  | .03(.03) | [-.02, .05] |
| PMP | | -.03(.03) | [-.09, .03] |  | **.11* (.05)** | [.02, .22] |  | -.03(.03) | [-.07, .02] |

N=314. The path coefficient in the model is standardized coefficient (*β*). Significant results are displayed in bold.* p < 0.05, ** p < 0.01, *** p < 0.001.

Supplementary Table 2. The significant direct and indirect effects of perceptions of successes and problems on intergenerational relationships and parents' feelings（Estimate(SE)）.

| Variables |  | DA | IA | NCIA | PCINA | RS | GS | PNF | PPF |
| --- | --- | --- | --- | --- | --- | --- | --- | --- | --- |
| PSCC | Direct effect |  | 0.21**  (0.08) | 0.21*  (0.10) |  |  | 0.31***  (0.09) |  | 0.20**  (0.07) |
|  | Indirect effect |  | -0.05^+^  (0.03) | -0.04*  (0.02) | 0.08*  (0.03) | 0.06*  (0.02) | 0.07*  (0.03) |  |  |
| PSHHPR | Direct effect | -0.33***  (0.09) | -0.21**  (0.08) | -0.21*  (0.09) |  |  |  | -0.28***  (0.07) | 0.41  (0.08) |
|  | Indirect effect |  | -0.13***  (0.03) | -0.11***  (0.03) | 0.20***  (0.05) | 0.12**  (0.04) | 0.14***  (0.04) |  |  |
| PSCP | Direct effect |  |  |  |  | -0.20**  (0.07) |  | -0.19***  (0.05) |  |
|  | Indirect effect |  | -0.05*  (0.02) | 0.04*  (0.02) | 0.06^+^  (0.03) |  |  |  |  |
| PSP | Direct effect | 0.25***  (0.07) | 0.29***  (0.07) | 0.28***  (0.07) |  |  |  | 0.27***  (0.07) |  |
|  | Indirect effect |  | 0.08**  (0.03) | 0.05*  (0.03) | -0.09*  (0.04) |  |  |  |  |
| PMP | Direct effect |  |  |  |  |  |  |  |  |
|  | Indirect effect |  |  |  |  |  |  |  |  |
| PFP | Direct effect |  |  | -0.15*  (0.07) |  |  |  | 0.16*  (0.07) |  |
|  | Indirect effect |  |  |  | -0.07^+^  (0.04) |  |  |  |  |

N=314. The path coefficient in the model is standardized coefficient. sig. is short for significant. +p<.01, * p < 0.05, ** p < 0.01, *** p < 0.001.
